# The importance of eating patterns for health-related quality of life among children aged 10–11 years in Alberta of Canada

**DOI:** 10.1038/s41598-022-23707-7

**Published:** 2022-12-03

**Authors:** Xiu Yun Wu, Arto Ohinmaa, Katerina Maximova, Paul J. Veugelers

**Affiliations:** 1 School of Public Health, University of Alberta, Edmonton Clinic Health Academy, 11405 87 Ave NW, Edmonton, AB T6G 1C9 Canada; 2 MAP Centre for Urban Health Solutions, St. Michael’s Hospital, 209 Victoria Street, 3Rd Floor, Toronto, ON M5B 1T8 Canada; 3 Dalla Lana School of Public Health, University of Toronto, Toronto, ON Canada

**Keywords:** Psychology, Diseases, Risk factors

## Abstract

Children with unhealthy eating behaviours are more likely to experience poor physical and mental health. Few studies have investigated the importance of eating patterns for health-related quality of life (HRQoL) among children. This study aimed to identify common eating patterns, and their associations with HRQoL among Canadian children. Data were collected from 9150 grade five students (aged 10–11 years) in repeat cross-sectional population-based surveys in Alberta, Canada. Students’ eating behaviours were analyzed using latent class analysis to identify the eating patterns. We applied multilevel multivariable logistic regression to examine the association of the eating patterns with HRQoL. We identified three groups of children with distinct eating patterns: eating healthy (52%), less healthy (31%) and unhealthy (17%). The first group had a higher proportion of students engaged in healthy eating behaviours. The unhealthy pattern group (third group) included a higher proportion of students with poor eating behaviours. Students’ eating behaviours in the second group were healthier than the third group but less healthy than the first group. Children with unhealthy and less healthy patterns were more likely to experience lower HRQoL than children with the healthy pattern. Health promotion programs effective in improving healthy eating patterns may not only reduce the risk for chronic diseases in the long term, but also improve the HRQoL in the short term.

## Introduction

An unhealthy eating behaviour is associated with a higher intake of saturated fat, processed foods, sugar and inadequate intake of healthful foods^1^. It has been documented that unhealthy eating behaviours such as skipping breakfast, eating meals in front of a television (TV), eating fast food and fried food are major risk factors for childhood and adolescence obesity and cardio-metabolic diseases^2–4^. Evidence shows that unhealthy eating behaviours are associated with a wide range of mental health problems such as depression, poor psychosocial well-being and low self-esteem among children and adolescents^5–7^. In contrast, healthy eating behaviours like eating meals with families and eating breakfast and meals regularly are associated with better mental health among children and adolescents^5,8^. Eating behaviours are also associated with diet quality among children and adolescents^9,10^. Research on the effects of diet and eating behaviours on health among children and adolescents has mainly focused on examinations of individual nutrients, specific food groups or aggregate measures of diet quality^3,4,11^. Few studies have analyzed the relationship between eating patterns (combinations of eating behaviours such as eating while watching TV, eating with family, skipping breakfast, choosing fast food, snacks or fried food) and health among children and adolescents^12,13^. Eating patterns provide information on cumulative influences of multiple eating behaviours on health outcomes that are not captured when eating behaviours are analyzed in isolation^14,15^.

Health-related quality of life (HRQoL) measures an individual’s overall health status as well as the health of underlying sub-dimensions: physical, psychological and social functioning and wellbeing^16^. Studies that examined the relationship of diet and eating behaviours with HRQoL among children and youth have mostly focused on the association between aggregate measures of diet quality or single eating behaviour and HRQoL^11^. Interpreting the effect of a single health-related behaviour on health often provides limited explanatory power for the association of interest as health-related behaviours generally co-occur to produce synergistic or combined effects on health indicators like HRQoL^15,17^. Prior research on families with adolescents has shown that multiple eating behaviours tend to cluster in ways that reflect meaningful heterogeneous eating patterns among individuals that influence their health outcomes^15^. There is some evidence that health-related behaviours patterns comprising diet, physical activity (PA) and/or sedentary behaviour are associated with overweight and obesity among children and adolescents^18,19^. However, there is a lack of research that examines how multiple eating behaviours in combination may influence health and HRQoL among children and adolescents. To the best of our knowledge, no studies have investigated the association of eating patterns with HRQoL among children that constitute multiple eating behaviours. Given that healthy eating habits are usually established in childhood and persist into later life^20^, understanding the impact of eating patterns on HRQoL is critical to inform intervention strategies that promote healthy eating, health-related quality of life and physical health among children.

The province of Alberta is the fourth largest province in Canada with a population of more than 4.54 million in 2022 compared to 3.87 million in 2012 with an annual population growth of 2.2% over the past decade (https://www.alberta.ca/population-statistics.aspx). The proportion of childen and adolescents aged less than 15 years was 19.0% in 2021 and 18.8% in 2011. The median households income after-tax was $83,000 in 2020 and $83,800 in 2011 (https://www12.statcan.gc.ca/census-recensement/stats/statgeo2021.cfm?Lang=E&Dguid=2021A000248&tid=0). The average number of children in census families with children was 1.9 in 2021 and 2011. Population census data in Alberta did not show a significant change over the past 10 years in terms of the general characteristics of children, such as the proportion of children among population, the average family income, number of children in a family.

The primary aim of this study was to identify eating patterns of children based on their eating behaviours, and to examine the associations between the eating patterns and health-related quality of life among children. The second aim was to characterize the identified eating patterns according to body weight status, diet quality and socio-demographic characteristics of children.

## Methods

### The REAL Kids Alberta surveys

The 2008, 2010 and 2012 Raising Healthy Eating and Active Living Kids in Alberta (REAL Kids Alberta) surveys aimed to evaluate a comprehensive initiative by Alberta Health and Wellness to promote healthy dietary and lifestyle behaviours and health among school children. Participants were grade five students who were primarily 10 and 11 years of age, and their parents. The survey began in 2008 and was repeated in 2010 and 2012. The survey employed a one-stage stratified random sampling design with a sampling frame that included all elementary schools in the province of Alberta with grade five students, with the exception of private schools (4.7% of all Albertan students), francophone schools (0.6%), on-reserve federal schools (2.0%), charter schools (1.7%) and colony schools (0.8%)^21,22^. Schools were stratified into three geographic regions: urban (cities of Calgary and Edmonton); towns (municipalities with population ≥ 40,000); and rural (municipalities with population less than 40,000). Schools were randomly selected within each of the strata to ensure proportional representation of schools from each geographic region^21,22^. The design and findings of the surveys were reported in more detail elsewhere^21–23^. In this study, we used the combined data of the 2008, 2010 and 2012 REAL Kids Alberta surveys. The survey included a student survey that was completed by students in the schools, and a home survey completed by their parents. The survey was administered to students during classroom time by trained assistants. The student survey included the validated Harvard Youth/Adolescent Food Frequency Questionnaire (YAQ)^24,25^ adapted version for Canadian children and youth, and the EQ-5D-Y descriptive system^26^. The YAQ included questions on nutrient intake, food items and eating behaviours. The home survey included information on children’s socio-demographic characteristics, including gender, place of residency (metropolitan, city, rural-town), household income, highest level of parental education and questions on children’s PA with and without a coach.

Written informed consent to participate in the surveys was provided by the participants’ legal guardians/next of kin, and all students provided assent. The Health Research Ethics Board of the University of Alberta and participating school boards approved all the surveys. The present study was approved by the Health Research Ethics Board of the University of Alberta. All methods were performed in accordance with the relevant national and international guidelines and regulations.

### Assessments of health-related quality of life

Health-related quality of life was measured by the EQ-5D-Y (youth), which was designed for children and youth aged between 8 and 18 years^26^. The EQ-5D-Y measure consists of a five-dimensional descriptive system asking whether children have (i) no problems, some problems or (iii) a lot of problems on: (a) walking; (b) looking after self; (c) doing usual activities; (d) having pain or discomfort; and (e) feeling worried, sad or unhappy, respectively. The instrument also includes a Visual Analogue Scale (VAS) which is anchored at 100 (best imaginable health) and 0 (worst imaginable health) to capture self-reported values of the overall health status in children. The EQ-5D-Y has been validated and used in many languages and countries^27^ (https://euroqol.org/eq-5d-instruments/eq-5d-y-about). In the present study, we used the five dimensions of the EQ-5D-Y descriptive system as the health outcomes.

### Assessments of eating patterns

The YAQ included questions on eating behaviours related to children’ dietary habits and meal regularity, and questions on various food items and nutrients consumed. The student survey included questions asking the frequency of buying snacks at schools like donuts, candy, chocolates, etc. In this study, we used 12 items on eating patterns that covered elements of meals and food groups: (1) eating breakfast, (2) bringing prepared lunch from home, (3) buying lunch at school, (4) buying snacks at school like donuts, candy, chocolates, etc., (5) fruit and vegetables’ intake, (6) eating supper with family, (7) eating supper in front of the TV, (8) eating supper alone, (9) eating supper ready-made, (10) eating fast food at restaurant, (11) eating fried food at home, (12) eating fried food outside home. The response options for the first three questions were “yes” or “no”. The fruit and vegetables intake was based on a number of daily servings. We dichotomized the fruit and vegetables consumption as follows: ≥ 6 vs. < 6 servings based on recommendations for fruit and vegetables for children aged 9–11 years according to the Health Canada’s Eating Well with Canada’s Food Guide^28^. The other eating behaviours were based on weekly occurrence and were categorized as follows: Eating supper with family: ≤ 2, 3–4 and ≥ 5 times per week; Eating supper in front of TV, Eat supper alone, Eating supper ready-made, and Eating at a fast food restaurant: < 1 time per month, 1–2 and ≥ 3 times per week; Eating fried food at home and outside home: < 1, 1–3 and 4–6 times or daily per week; Buying snacks at school: < 1, 1–2 and ≥ 3 times per week. The 12 items were used as they represent eating habits and meal regularity, and previous studies have reported that the eating behaviours were associated with health among children and youth^7,15,29^.

### Socio-demographic characteristics

Socio-demographic characteristics of the students included children’ gender, residential area, highest level of parental education and household income. Residency was classified as urban (metropolitan and city) and rural (rural-town) area. Parental educational attainment was categorized as secondary school or lower, college, and university or above. Annual household income was categorized into four levels: less than or equal to $50,000, $50,001–$75,000, $75,001–$100,000 and > $100,000.

### Assessments of body weight status, diet quality and physical activity

Standing height of the student was measured by a research assistant to the nearest 0.1 cm, and body weight was measured to the nearest 0.1 kg on calibrated digital scales. We adopt the age- and gender-specific body mass index (BMI) cut-off points for children established by the International Obesity Task Force^30^. The body weight status is categorized as normal weight, overweight and obese.

On the basis of children’ nutrient intake and dietary information from the YAQ^24^ and the Canadian Nutrient Files^31^, we calculated overall diet quality using the Diet Quality Index-International score (DQI-I)^32^. The DQI-I scores range between 0 and 100, with higher scores indicating better diet quality. The overall DQI-I score was categorized into tertiles for analysis.

The REAL Kids Alberta home survey included questions adopted from the National Longitudinal Survey for Children and Youth in Canada on children’ playing sports or doing physical activities with and without a coach^33^. The questions were categorized as weekly times spent on the physical activities: Never, 1–3 times a week, and ≥ 4 times a week.

### Statistical analysis

Descriptive analyses included frequency distributions of the items used for eating patterns, the EQ-5D-Y dimensions, body weight status, diet quality index tertiles, PA and the socio-demographic characteristics of children.

To identify homogeneous groups (i.e., latent classes) of children with similar patterns of eating behaviours, latent class analysis (LCA) was used. LCA is data driven statistical method that identifies unobserved homogeneous groups of respondents with similar patterns of responses on multiple observed item indicators^34^. The major advantage of LCA over conventional cluster analysis is that LCA is a model-based method that can provide fit statistics for choosing the most appropriate model for the data^35^. In addition, LCA allows covariates or a distal outcome to be included in the model to examine their relationship with the latent class membership and thus providing results of both the latent classes and their associations with the covariates or distal outcomes directly^34^. Since LCA method is based on the structure of the data, results can be generalized to target populations and provide important implications for health intervention strategies.

Indicators used for the LCA included the aforementioned 12 eating behaviour items, which were all categorical variables. Two items (eating supper with family, fruit and vegetables) were reverse coded in the analysis such that a higher score indicated poorer eating behaviour or lower intake of fruit and vegetables. The variable coding for the eating behaviour questions in the latent class analysis is shown in the supplementary information (Table S1). A series of LCA models were fitted in order to choose the model with the optimal number of classes. First, a one class model was fitted, and then successive models were estimated with an increase in the number of classes, up to five classes. The multiple-class (e.g., k classes) models were compared with the k-1 class model based on the fit statistics’ criteria. The model fit criteria included the Bayesian Information Criterion (BIC), the sample size-adjusted BIC (aBIC) and the Lo-Mendell-Rubin adjusted likelihood ratio test (LMRALRT)^36^. A lower value of BIC and aBIC indicates a better fit of the k-classes model compared with the k-1 classes model^36^. A non-significant p value (p > 0.05) of the LMRALRT for a k classes model indicates a better fit of the k-1 classes model relative to the k classes model. Classification accuracy of the classes was assessed by entropy ranging between 0 and 1. A higher entropy indicates better classification. The model selection was based on a better overall fit and substantive meaningful interpretation of the latent classes. The latent class model was estimated with maximum likelihood estimation with robust standard errors (MLR), and accommodated population survey design (e.g., school clusters)^34^.

First, multilevel multinomial logistic regression model was fitted to the data to examine the relationships of the eating patterns with HRQOL, body weight status, diet quality and socio-demographic characteristics, respectively. Second, separate multilevel logistic regression models were fitted to assess the relationship of the eating patterns with each dimension of the EQ-5D-Y adjusting for confounding influence of the socio-demographic variables, body weight status and diet quality. Missing values for residency, parental education and household income, body weight status and PA were considered as separate covariate categories in the regression models but the estimates are not presented. All analyses were weighted to represent provincial estimates of the grade five students in Alberta.

We used the merged 2008, 2010 and 2012 survey data as very few number of students responded to “yes” for skipping breakfast and buying lunch at school in single year survey (e.g., 98 in 2008 and 71 in 2012 for skipping breakfast; 59 in 2012 for buying lunch at school). Simulation studies have shown that large sample contribute to improved outcomes in identifying classes^36^.

The latent class analyses were conducted using Mplus version 8^34^. All other analyses were conducted using the statistical software of Stata/IC 15 (College Station, TX: StataCorp LLC).

The present study was reported adhering to the STROBE statement guideline for cross-sectional studies (https://www.strobe-statement.org/).

## Results

### Student characteristics and distribution of HRQoL

In total, 3421, 3398 and 2337 students completed the REAL Kids Alberta surveys in 2008, 2010 and 2012, respectively. After excluding students who provided incomplete information on all 12 eating behaviours (n = 6), a total of 9150 students were included in analysis.

Table 1 shows the frequency distributions of socio-demographic characteristics, body weight, physical activity and diet quality of children. Of the total respondents, 51.5% were girls; and 19.73%, 7.59% were overweight and obese respectively.

**Table 1:** Frequency distributions of socio-demographic characteristics, body weight, physical activity and diet quality among of grade five students participating in the 2008, 2010 and 2012 Real Kids Alberta surveys in Canada.

Variable	Percentage (%)
**Gender (n = 9149)**	
Girls	51.48
Boys	48.52
**Residence (n = 9150)**	
Urban	62.55
Rural	37.43
Missing	0.02
**Parental education (n = 9150)**	
Secondary school or lower	24.51
College	36.90
University or above	33.34
Missing	5.25
**Household income ($CAN/per year) (n = 9150)**	
≤ $50,000	16.71
$50,001-$75,000	11.95
$75,001-$100,000	14.01
> $100,000	25.47
Do not know/not to answer/missing	31.86
**Body weight status (n = 9150)**	
Normal weight	71.10
Overweight	19.73
Obese	7.59
Missing	1.59
**Physical activity without a coach (n = 9150)**	
Never	16.48
1–3 times/week	44.26
≥ 4 times/week	36.29
Missing	2.96
**Physical activity with a coach (n = 9150)**	
Never	33.25
1–3 times/week	46.92
≥ 4 times/week	16.72
Missing	3.11
**DQI-I (n = 9141)**	
Lowest tertile	–
Middle tertile	–
Highest tertile	–

For the EQ-5D-Y dimensions, the observations with “some problems” and “a lot of problems” were combined to one group as very few students answered “a lot of problems” (from 0.38% to 3.5%). The prevalence of having “some or a lot of problems” in the EQ-5D-Y dimensions was relatively high for “having pain or discomfort” (44.99%) and “feeling worried, sad or unhappy” (34.85%), whereas the prevalence of problems on other dimensions was relatively low (walking: 8.85%; looking after self: 5.37%; doing usual activities: 11.32%). Of the students who had complete information on the five EQ-5D-Y dimensions (n = 9131), 39.45% (n = 3569) did not report any problems, and the remaining students reported having “some or a lot of problems” on one (29.45%, n = 2673), two (20.84%, n = 1943), three (7.31%, n = 673), four (2.52%, n = 234) and five (0.43% , n = 39) dimensions, respectively.

### Groupings of the eating patterns

The fit statistics for the latent class models with one- to 5-classes are provided in the supplementary information (Table S2). The BIC and the sample size-adjusted BIC decreased from one- to 5-class models. The LMRALRT p-value was 0.0235 (< 0.05) for the 3-class model, 0.1087 (> 0.05) for the 4-class model and 0.5121 (> 0.05) for the 5-class model, respectively. The 4- and 5-class models have a class with a very small sample size (5%, 4% respectively). The test statistic of LMRALRT and the interpretation of the classes favored the 3-class model, thus the 3-class model was selected as the best parsimonious model and was used in the subsequent analyses.

**Figure 1:**
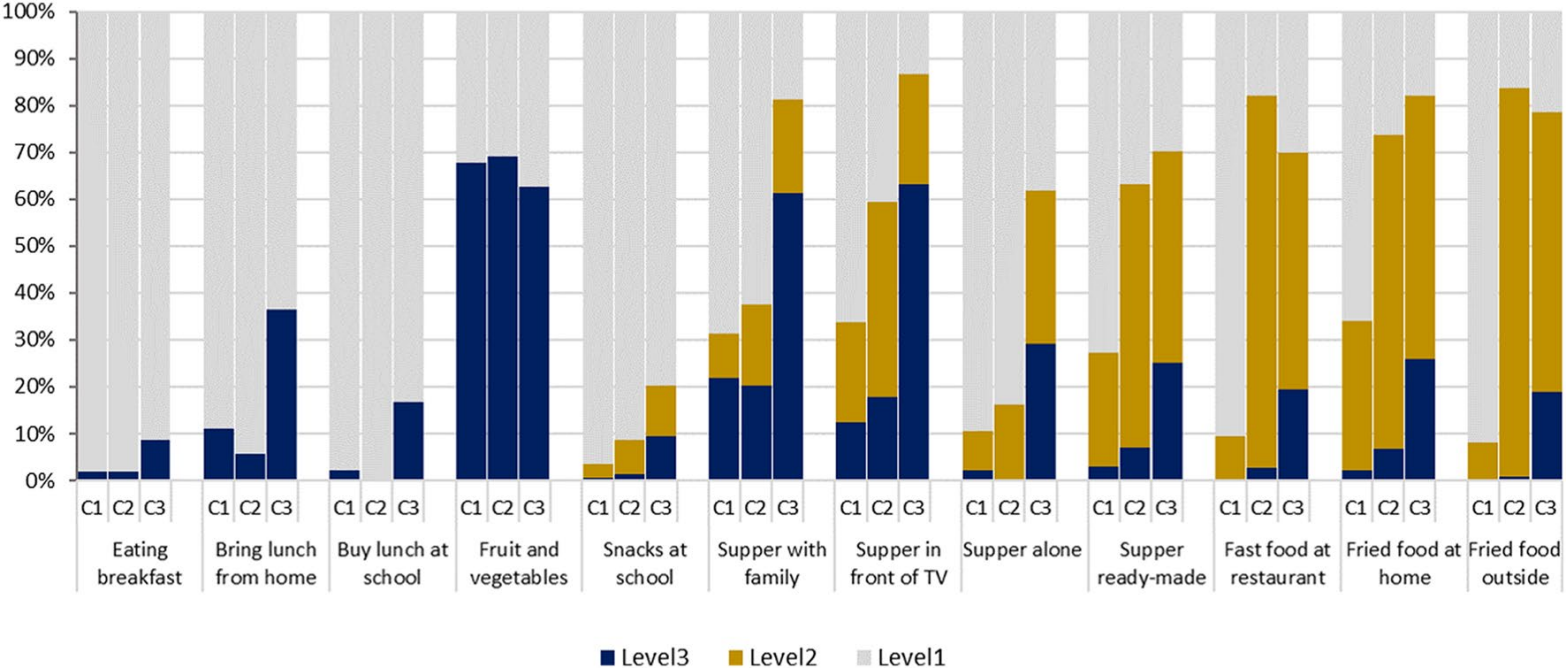
Within class item response percentages of the eating behaviours. C1-class 1 (healthy), C2-class 2 (less healthy), C3-class 3 (unhealthy). The item response level 1 to level 3 for the eating items were shown below. The items with three-categories: (a) Fast food at restaurant, Supper in front of TV, Supper alone, Supper ready-made: Level 3: ≥ 3 times per week; Level 2: 1–2 times per week; Level 1: less than 1 time per month; (b) Supper with family: Level 3: ≤ 2 times per week; Level 2: 3–4 times per week; Level 1: ≥ 5 times per week; (c) Eating fried food at home, Eating fried food out: Level 3: 4–6 times or daily per week; Level 2: 1–3 times per week; Level 1: Less than 1 time per week; (d) Buying snacks at school: Level 3: ≥ 3 times per week; Level 2: 1–2 times per week Level 1: Never/ < Once per week. The items with two-categories: (a) Eating breakfast, Bring lunch from home: Level 3: No, Level 1: Yes; (b) Buying lunch at school: Level 3: Yes, Level 1: No; (c) Fruit and vegetable intake: Level 3: < 6 daily servings, Level 1: ≥ 6 daily servings.

Table 2 depicts the response percentages for the 12 eating items in total sample and in the subgroups of the latent classes. The within class item response percentages of the eating behaviours by classes are graphically presented in Fig. 1. Approximately half of children (52%) were in the first grouping with an eating pattern labeled as “healthy” relative to the other two classes. Children in this grouping had the lowest percentage of skipping breakfast (1.79%) among all classes, a lower percentage of buying lunch at school (2.13%), a higher percentage of bringing lunch from home (88.93%), and slightly lower intake of fruit and vegetables relative to class 3, and the highest percentage of eating supper with family ≥ 5 times per week (68.76%). The first grouping had lowest percentages of children with poor eating (e.g., response level 3 and level 2 in Fig. 1) on the remaining seven items (buying snacks at school, eating supper in front of TV, supper alone, supper ready-made, eating fried food at home and outside and eating at a fast-food restaurant). No children in the first grouping reported eating at a fast-food restaurant and eating fried food outside home ≥ 3 times per week (Table 2).

**Table 2:** Item response percentages (%) of the eating behaviors overall and by the latent class, grade five students participating in the 2008, 2010 and 2012 Real Kids Alberta surveys in Canada.

Variables	Class 1 (Healthy)	Class 2 (Less healthy)	Class 3 (Unhealthy)	Total sample
Class size	4819	2870	1461	9150
**Eating breakfast (n = 9107)**				
Yes	98.21	98.05	91.24	96.95
No	1.79	1.95	8.76	3.05
**Bringing prepared lunch from home (n = 9143)**				
Yes	88.93	94.23	63.42	86.14
No	11.07	5.77	36.58	13.86
**Buying lunch at school (n = 9143)**				
No	97.87	100	83.14	95.97
Yes	2.13	0.00	16.86	4.03
**Fruit and vegetables intake (n = 9141)**				
≥ 6 daily servings	32.10	30.76	37.46	32.62
< 6 daily servings	67.90	69.24	62.54	67.38
**Buying snacks at school (n = 9037)**				
Never/ < once per week	96.59	91.39	79.78	92.05
1–2 times per week	2.74	7.29	10.85	5.56
≥ 3 times per week	0.67	1.32	9.37	2.39
**Eating supper with family (n = 9052)**				
≥ 5 times per week	68.76	62.45	18.63	58.15
3–4 times per week	9.29	17.40	19.96	13.64
≤ 2 times per week	21.95	20.15	61.40	28.21
**Eating supper in front of TV (n = 9072)**				
< 1 time per month	66.30	40.62	13.21	49.16
1–2 times per week	21.40	41.42	23.52	27.97
≥ 3 times per week	12.30	17.95	63.26	22.86
**Eat supper alone (n = 9080 )**				
< 1 time per month	89.36	83.84	38.25	78.82
1–2 times per week	8.44	16.16	32.54	15.00
≥ 3 times per week	2.20	0.00	29.21	6.18
**Eating supper ready-made (n = 9090)**				
< 1 time per month	72.75	36.90	29.88	54.22
1–2 times per week	24.20	55.97	45.04	37.67
≥ 3 times per week	3.05	7.13	25.09	8.12
**Eating at a fast food restaurant (n = 9089 )**				
< 1 time per month	90.67	17.98	30.01	57.64
1–2 times per week	9.33	79.37	50.49	38.16
≥ 3 times per week	0.00	2.65	19.50	4.20
**Eating fried food at home (n = 9106)**				
< 1 time per week	65.90	26.38	17.82	45.32
1–3 times per week	31.84	67.00	56.35	46.98
4–6 times or daily per week	2.26	6.62	25.83	7.70
**Eating fried food outside home (n = 9088)**				
< 1 time per week	91.91	16.40	21.39	56.32
1–3 times per week	8.09	82.68	59.64	40.10
4–6 times or daily per week	0.00	0.92	18.97	3.58

Class 1: Healthy eating pattern: lowest percentage of breakfast skipping, higher percentage of bringing lunch from home, and lower percentage of buying lunch at school, and lower fruit and vegetables intake ≥ 6 daily servings (relative to class 3); highest percentage of eating supper with family, lowest percentage of buying snacks at school, lowest percentage of eating supper in front of TV/supper alone/supper ready-made; lowest rate of eating at fast food restaurant, eating fried food at home and outside.

Class 2: Less healthy eating pattern: lower percentage of breakfast skipping than class 3, highest percentage of bringing lunch from home, no buying lunch at school, lowest percentage of fruit and vegetables intake ≥ 6 daily servings; highest percentage of eating at fast food restaurant and eating fried food outside greater than 1 times per week; relative to the other two classes, class 2 had moderate level of buying snacks at school, eating supper with family, eating supper in front of TV/supper alone/supper ready-made, and eating fried food at home.

Class 3: Unhealthy eating pattern: highest percentage of breakfast skipping and buying lunch at school, lowest percentage of bringing lunch from home, highest percentage of fruit and vegetables intake ≥ 6 daily servings. For the items with three response levels, class 3 had highest percentage of children reporting the poorest levels (level 3 in Fig. 1) on all the 8 items, and had highest percentage of reporting both level 2 and level 3 on 6 items (buying snacks at school, eating supper with family, eating supper in front of TV/supper alone/supper ready-made and eating fried food at home).

The second grouping represents about one third of children (31%) with a pattern of “less healthy” relative to the other two classes (Fig. 1). In general, the second grouping (class 2) was between class 1 and 3 with respect of the percentage of children who responded to the less and least healthy levels of the eating items, where the percentages were lower than the third but higher than the first grouping for most of the items. The percentage of responding to the most unhealthy level was slightly lower for the questions of bringing lunch from home, buying lunch at school and eating supper alone, and slightly higher but not statistically significant for fruit and vegetable intake than the first grouping. (Table 2, Fig. 1).

The third grouping included 17% of children with an eating pattern labelled as “unhealthy”. Among the three groupings, the third grouping had the highest proportions of children responding to the unhealthy levels (e.g., level 3 for the items with 3 categories and level 2 for the dichotomous items in Fig. 1) on all the eating items with only an exception for fruit and vegetable intake.

### Associations of the eating patterns with HRQoL and other characteristics

Table 3 shows the frequency distribution of the EQ-5D-Y dimensions, the socio-demographic characteristics, body weight status, PA and diet quality by the eating pattern (latent class), and the likelihood of group membership by each of the explanatory variables. Children in grouping 2 and grouping 3 (relative to grouping 1) had a higher prevalence of experiencing some or a lot of problems in each of the EQ-5D-Y dimensions. Children lived in families with higher household income were less likely to engage in poor eating patterns (grouping 2 and 3), and children with the highest parental education (university or above) relative to the lowest parental education (secondary school or lower) were less likely to be in the grouping of unhealthful eating pattern. Boys were more likely to be assigned in the grouping of unfavourable eating pattern than girls (OR = 1.36, 95% CI: 1.20, 1.53 for class 3, p < 0.001). Children who were obese (OR = 1.38, 95% CI: 1.01, 1.89) and physically inactive were more likely to experience multiple poor eating behaviours. Children in the highest tertile of diet quality (versus lowest tertile) had a lower likelihood of being in grouping 3, and children in the middle tertile of the diet quality had a higher likelihood of being in grouping 2.

**Table 3:** Frequency distributions of the EQ-5D-Y dimensions, the socio-demographic characteristics, body weight, physical activity and diet quality by the class, and the odds of their class membership, grade five students participating in the 2008, 2010 and 2012 Real Kids Alberta surveys in Canada.

Variables	Distribution (%)			The odds of class membership			
	Class 1	Class 2	Class 3	Class 2		Class 3	
				OR (95% CI)	*P value*	OR (95% CI)	*P value*
**EQ-5D-Y dimensions**							
**Walking**							
No problems	92.93	90.95	86.23	1.0		1.0	
Some or a lot of problems	7.07	9.05	13.77	1.28 (1.04, 1.57)	**0.018**	2.05 (1.68, 2.51)	** < 0.001**
**Looking after self**							
No problems	96.04	93.75	91.97	1.0		1.0	
Some or a lot of problems	3.96	6.25	8.03	1.56 (1.23, 1.98)	** < 0.001**	2.06 (1.54, 2.75)	** < 0.001**
**Doing usual activities**							
No problems	91.30	87.55	82.91	1.0		1.0	
Some or a lot of problems	8.70	12.45	17.09	1.49 (1.26, 1.77)	** < 0.001**	2.16 (1.72, 2.71)	** < 0.001**
**Having pain or discomfort**							
No problems	60.18	51.70	45.50	1.0		1.0	
Some or a lot of problems	39.82	48.30	54.50	1.43 (1.27, 1.61)	** < 0.001**	1.83 (1.56, 2.16)	** < 0.001**
**Feeling worried, sad or unhappy**							
No problems	69.38	63.82	54.92	1.0		1.0	
Some or a lot of problems	30.62	36.18	45.08	1.30 (1.17, 1.45)	** < 0.001**	1.89 (1.64, 2.18)	** < 0.001**
**Other variables**							
**Gender**							
Girls	53.07	52.04	45.75	1.0		1.0	
Boys	46.93	47.96	54.25	1.06 (0.93, 1.20)	0.382	1.36 (1.20, 1.53)	** < 0.001**
**Residence**							
Urban	61.71	61.07	67.77	1.0		1.0	
Rural	38.29	38.93	32.23	1.01 (0.86, 1.17)	0.943	0.75 (0.56, 1.00)	**0.047**
**Parental education**							
Secondary school or lower	22.43	26.32	35.54	1.0		1.0	
College	39.11	38.71	38.88	0.88 (0.76, 1.01)	0.074	0.66 (0.57, 0.76)	** < 0.001**
University or above	38.46	34.97	25.58	0.86 (0.75, 1.00)	**0.050**	0.47 (0.35, 0.63)	** < 0.001**
**Household income** ($CAN/per year)							
≤ $50,000	18.67	24.61	42.08	1.0		1.0	
$50,001-$75,000	17.44	16.75	19.29	0.75 (0.60, 0.95)	**0.016**	0.51 (0.41, 0.65)	** < 0.001**
$75,001-$100,000	21.81	19.96	17.93	0.76 (0.63, 0.92)	**0.006**	0.40 (0.31, 0.51)	** < 0.001**
> $100,000	42.09	38.68	20.70	0.82 (0.67, 1.00)	**0.051**	0.25 (0.20, 0.32)	** < 0.001**
**Body weight status**							
Normal weight	73.31	72.14	69.24	1.0		1.0	
Overweight	19.76	20.00	21.01	0.97 (0.84, 1.13)	0.724	1.07 (0.89, 1.29)	0.475
Obese	6.94	7.86	9.75	1.07 (0.84, 1.37)	0.581	1.38 (1.01, 1.89)	**0.041**
**Physical activity without a coach**							
Never	14.80	18.02	21.75	1.0		1.0	
1–3 times/week	46.83	44.67	43.64	0.83 (0.70, 0.97)	**0.020**	0.67 (0.53, 0.85)	**0.001**
≥ 4 times/week	38.37	37.32	34.60	0.85 (0.71, 1.02)	0.089	0.66 (0.51, 0.85)	**0.001**
**Physical activity with a coach**							
Never	29.67	33.24	50.35	1.0		1.0	
1–3 times per week	51.13	49.67	37.98	0.94 (0.81, 1.09)	0.417	0.47 (0.40, 0.57)	** < 0.001**
≥ 4 times per week	19.20	17.09	11.67	0.90 (0.77, 1.04)	0.150	0.40 (0.32, 0.50)	** < 0.001**
**DQI-I**							
Lowest tertile	32.22	31.34	36.39	1.0		1.0	
Middle tertile	30.43	37.07	33.07	1.31 (1.14, 1.51)	** < 0.001**	1.01 (0.84, 1.20)	0.940
Highest tertile	37.35	31.59	30.54	0.91 (0.78, 1.05)	0.177	0.75 (0.63, 0.90)	**0.002**

Class 1: Healthy, Class 2: Less healthy, Class 3: Unhealthy. Class 1 was the reference group in the multinomial logistic regression. Bold *P* values indicate a statistically significance (p < 0.05).

*OR* odds ratio, *CI* confidence interval, *P* p value.

Table 4 shows the associations between the eating patterns and HRQoL. After adjusting for covariates, children in the unhealthy and less healthy pattern groups (relative to the healthy pattern) had a higher likelihood of reporting health problems on each of the EQ-5D-Y dimensions. Moreover, the odds of experiencing problems on each dimension in grouping 3 was higher than the likelihood in grouping 2 (e.g., OR (95% CI): Walking: 2.00 (1.61, 2.48) for class 3, 1.28 (1.06, 1.56) for class 2; Feeling worried, sad or unhappy: 1.85 (1.59, 2.15) for class 3, 1.28 (1.15, 1.42) for class 2), suggesting a dose–response association between the eating patterns and HRQoL among children.

**Table 4:** The odds of reporting some or a lot of problems in the EQ-5D-Y dimensions by the eating patterns, grade five students participating in the 2008, 2010 and 2012 Real Kids Alberta surveys in Canada.

Variables	Walking		Looking after self		Doing usual activities		Having pain or discomfort		Feeling worried, sad or unhappy	
	OR (95% CI)	*P*	OR (95% CI)	*P*	OR (95% CI)	*P*	OR (95% CI)	*P*	OR (95% CI)	*P*
**Eating patterns (reference: Class 1)**										
Class 2	1.28 (1.06, 1.56)	**0.012**	1.53 (1.20, 1.96)	**0.001**	1.45 (1.22, 1.72)	** < 0.001**	1.40 (1.25, 1.57)	** < 0.001**	1.28 (1.15, 1.42)	** < 0.001**
Class 3	2.00 (1.61, 2.48)	** < 0.001**	1.84 (1.34, 2.52)	** < 0.001**	1.96 (1.56, 2.46)	** < 0.001**	1.82 (1.55, 2.14)	** < 0.001**	1.85 (1.59, 2.15)	** < 0.001**
**Gender (reference: Girls)**										
Boys	0.96 (0.81, 1.13)	0.628	1.29 (1.05, 1.57)	**0.013**	0.93 (0.80, 1.09)	0.396	0.96 (0.88, 1.05)	0.411	0.64 (0.58, 0.72)	** < 0.001**
**Residence (reference: Urban)**										
Rural	1.20 (1.00, 1.44)	0.051	1.11 (0.88, 1.39)	0.389	1.37 (1.17, 1.60)	**0.001**	1.16 (1.05, 1.29)	**0.005**	1.01 (0.90, 1.12)	0.900
**Parental education (reference: Secondary school or lower)**										
College	1.09 (0.91, 1.32)	0.347	0.98 (0.77, 1.25)	0.892	0.94 (0.78, 1.13)	0.481	0.97 (0.87, 1.07)	0.512	1.04 (0.91, 1.19)	0.573
University or above	0.86 (0.68, 1.10)	0.225	1.39 (1.02, 1.90)	**0.040**	0.91 (0.73, 1.12)	0.352	0.92 (0.81, 1.05)	0.240	1.00 (0.87, 1.14)	0.953
**Household income ($CAN/per year) (reference: ≤ $50,000)**										
$50,001-$75,000	1.18 (0.90, 1.55)	0.230	1.35 (0.90, 2.05)	0.150	1.48 (1.15, 1.90)	**0.002**	1.28 (1.07, 1.52)	**0.006**	1.07 (0.89, 1.27)	0.474
$75,001-$100,000	1.06 (0.80, 1.39)	0.704	1.45 (1.04, 2.03)	**0.027**	1.19 (0.93, 1.54)	0.173	1.13 (0.95, 1.34)	0.181	1.03 (0.86, 1.22)	0.777
> $100,000	1.04 (0.79, 1.36)	0.801	1.10 (0.80, 1.49)	0.563	1.06 (0.86, 1.31)	0.561	1.08 (0.93, 1.25)	0.307	1.08 (0.93, 1.26)	0.311
**Body weight status (reference: normal weight)**										
Overweight	1.02 (0.84, 1.24)	0.839	0.97 (0.76, 1.24)	0.805	1.14 (0.94, 1.37)	0.182	1.07 (0.95, 1.21)	0.281	1.11 (1.00, 1.25)	0.057
Obese	1.21 (0.93, 1.59)	0.162	1.65 (1.20, 2.26)	**0.002**	1.35 (1.07, 1.70)	**0.013**	1.02 (0.86, 1.22)	0.785	1.26 (1.05, 1.52)	**0.013**
**Physical activity without a coach (reference: never)**										
1–3 times per week	0.76 (0.60, 0.96)	**0.022**	0.63 (0.46, 0.86)	**0.003**	0.72 (0.56, 0.92)	**0.008**	0.78 (0.66, 0.91)	**0.001**	0.94 (0.82, 1.07)	0.332
≥ 4 times per week	0.64 (0.51, 0.81)	** < 0.001**	0.54 (0.39, 0.74)	** < 0.001**	0.59 (0.46, 0.76)	** < 0.001**	0.75 (0.65, 0.88)	** < 0.001**	0.70 (0.60, 0.81)	** < 0.001**
**Physical activity with a coach (reference: never)**										
1–3 times per week	0.92 (0.76, 1.11)	0.382	0.67 (0.52, 0.86)	**0.002**	0.76 (0.64, 0.90)	**0.002**	1.07 (0.96, 1.18)	0.229	0.89 (0.80, 1.00)	**0.041**
≥ 4 times per week	0.87 (0.68, 1.11)	0.257	0.52 (0.37, 0.72)	** < 0.001**	0.56 (0.44, 0.72)	** < 0.001**	0.88 (0.76, 1.01)	0.078	0.67 (0.58, 0.79)	** < 0.001**
**DQI-I (reference: lowest tertile)**										
Middle tertile	0.84 (0.69, 1.03)	0.090	0.92 (0.73, 1.16)	0.459	0.89 (0.75, 1.06)	0.186	0.98 (0.87, 1.09)	0.668	1.07 (0.96, 1.19)	0.234
Highest tertile	0.80 (0.65, 0.99)	**0.038**	0.69 (0.54, 0.88)	**0.003**	0.73 (0.61, 0.88)	**0.001**	0.90 (0.81, 1.00)	**0.046**	1.00 (0.88, 1.13)	0.955

*P*: p value; bold p values indicate a statistically significance (p < 0.05). The regression model for each EQ-5D-Y dimension adjusted for children’ gender, residency, highest level of parental education, household income, body weight status, PA and diet quality.

*OR* odds ratio, *CI* confidence interval.

## Discussion

In this study, we identified three eating patterns among children who were primarily early-adolescents. Children with a healthy eating pattern (grouping 1) were the largest group and were more likely to report healthier eating behaviours on most of the eating items than the other groupings, and had slightly lower intake of fruit and vegetables (defined as equal or greater than 6 daily servings) than grouping 3. Children with unhealthy eating pattern (grouping 3) had a dominant poorest behaviouron most of eating items than the other two groupings, and a slightly higher intake of fruit and vegetables relative to grouping 1. The present study revealed that children engaged in unhealthful or less favourable eating patterns had significantly more problems on each of the EQ-5D-Y dimensions. We also observed that the unhealthy eating pattern is associated with higher prevalence of obesity, poor diet quality and lower PA among children. The results in this study strengthen the research evidence that unhealthy eating patterns are associated with lower health status among children and youth. Early-adolescence is a crucial period to develop health problems, and also a period to establish healthy lifestyle behaviours^1^. Given that children’ eating behaviours can persist into adults^20^, it is important to investigate how eating patterns impact their health and HRQoL to provide scientific reference information for public health intervention programs among children.

To our knowledge, this is the first study that applied a latent class analysis to identify eating patterns in children based on their reporting of multiple eating behaviours. LCA has been increasingly used to examine the clustering (or patterns) of diet and other health behaviours, including diet quality or dietary behaviour, PA and sedentary behaviour among children, adolescents and young adults^18,19^. Studies that have used LCA to characterize eating patterns based on multiple eating behaviours are scant^15^. We identified only one study that used LCA and evaluated adolescents’ family eating habits using family eating behaviours, such as eating large meals^15^. Further, no studies have applied LCA to understand the clustering of multiple eating behaviours considering simultaneously (a) the contextual factors (e.g., eating with families, or at fast food restaurant) and (b) meal types (e.g., breakfast and supper) among children.

The findings in this study support the notion that school age children have a variety of eating patterns (e.g., eating healthy or poor , and poor eating habits tend to cluster together to influence children’s mental and physical health^15,17,19^. The present study also revealed that less healthy and unhealthy eating styles are associated with more health problems in all five EQ-5D-Y dimensions of HRQoL, and there is a dose–response gradient in this relationship. Particularly, the finding of the dose–response association between the poor dietary patterns and lower HRQoL corroborates the evidence that eating behaviours can act together to yield synergistic effects on health status^15,17,19^. These findings are also in line with the previous studies that evaluated the influence of a single eating behaviour on HRQoL, showing that unhealthy eating behaviours such as eating fast food or takeaway food from restaurants and skipping breakfast were associated with lower HRQoL among children and youth^12,13^.

Our findings reveal a novel relationship of eating patterns with obesity and diet quality in children. We observed that the unhealthy eating pattern is related to a higher prevalence of childhood obesity and poor diet quality. The findings are consistent with some studies showing that unfavourable dietary patterns like eating fast foods and snacks, skipping meals and eating alone were associated with overweight and obesity, and poor diet quality, while a healthy dietary pattern was associated with better diet quality among children and adolescents^2,9,10,29,37^. Additionally, the finding that lower levels of physical activity were associated with unhealthy eating patterns is in line with other studies demonstrating that low physical activity correlates with poor dietary patterns or behaviours (e.g., higher fast food consumption, lower intake of fruit and vegetables)^29,37^.

The exact mechanisms through which different eating patterns impact HRQoL and associated health-related factors are unclear. Several possible explanations have been proposed. Unhealthy eating patterns, characterized as reduced intake of healthy foods (e.g., fruit and vegetables, fish, fibre, milk) and increased intake of unhealthy foods (e.g., fast and fried foods, processed foods, desserts) high in saturated fat and sugar, may lead to nutrient deficiencies^1^. Poor diet with insufficient nutrients can compromise body immune functioning among children^38^, resulting in increased risk of poor health, including HRQoL, physical and mental health^1,11^. Eating behaviours such as eating while watching TV, eating meals alone and eating without family may contribute to decreased communications and interactions with family and peers, thus increasing the feelings of loneliness and social isolation^5,7^. Previous studies have also reported possible pathways underlying the relationship between an unhealthy eating pattern and childhood obesity^3^. Future research would warrant to confirm the mechanisms for the relationships between eating patterns and HRQoL, obesity and other associated factors observed in this study.

The finding that boys are more likely than girls to be engaged in unhealthy eating behaviours corroborates previously published evidence^3,39^. For example, we observed that boys were more likely than girls to report eating supper in front of TV, eating fast food and fried food, which is consistent with previous studies showing that boys had a higher likelihood of eating calorie-rich snacks and fast food, and eating meals while watching TV than girls^3,39^. The observation that lower household income and lower parental education were associated with unhealthy eating patterns is consistent with previous studies showing that unhealthy dietary patterns (e.g., consumption of fast food) were related to lower socio-economic status and lower education levels of parents among children and adolescents^37,39^. The relationships of the estimated eating patterns with the socio-demographic characteristics and other health-related factors are mainly within the expectations, supporting a discriminant validity of the estimated eating patterns in identifying health differences by the socio-demographic and health-related variables.

This study has several strengths. We used a large random sample representative of the population of school children aged primarily 10–11 years in the province of Alberta. Population-based data are likely to exhibit substantial heterogeneity in patterns of health-related behaviours, which are well suited for the LCA. In the analysis of HRQoL, the large sample allowed us to adjust for important covariates such as socio-demographic variables, body weight status, PA and diet quality, thus enabling more robust results. In addition, the validated food frequency questionnaire (YAQ) for children and adolescents included information of both diet intakes and the related eating behaviours, which provided a unique opportunity to examine clustering patterns of eating behaviours using LCA and test their associations with the HRQoL.

One of the limitations of this study is the cross-sectional design, which precludes inference about causal or temporal relations of the eating behaviour patterns with HRQoL and other health indicators. Future research should consider analyses of longitudinal associations, which will help discover the temporal relationship between the eating patterns and health status among children. Experimental studies like randomized controlled trials targeting eating behaviour interventions among children are needed to better elucidate causal relationship between the eating patterns and HRQoL. We used the survey data from the province of Alberta, the present findings cannot be generalized to other provinces or the whole population in Canada. In addition, the data were collected 10 year ago, thus the results may not accurately represent the present status as children’s eating behaviours and health may change over time especially during the COVID-19 pandemic. Our results may underestimate the effect of poor eating patterns on health at present since recent studies have reported that children and adolescents appear to consume more snacks, fried foods and processed foods and engage in more sedentary activities during the COVID-19 pandemic than before the pandemic^40–42^. Research in Alberta has also demonstrated that Canadian children and youth have poor diets, and do not meet the Canada's Food Guide recommendations^43^. The recent study findings are largely consistent with our results showing that children had a low consumption of fruit and vegetable, increased time on eating in front of TV and eating without parents. Future research is needed to confirm the findings in this study. Studies that collect data of pre- and post-the pandemic would help to further analyze temporal changes of eating behaviours and their relationships with health during adolescence.

Investigating the association between dietary-related behavioral patterns and HRQoL is still understudied in child and adolescent populations, and it is important to take into account the effects of both individual behaviours and their combined patterns on the health outcomes. The findings in this study have important implications for health intervention strategies and future research among children. The intervention programs aimed at improving children’s HRQoL should consider the needs of sub-populations with different eating patterns and place greater emphasis on promoting overall eating patterns rather than single behaviours. The intervention strategies to enhance children’s health and HRQoL could consider promoting healthy eating environments both at schools and at home. The interventions should work to encourage and increase more opportunities for children to eat with families and eat regular and healthier foods, to reduce poor eating behaviours like skipping breakfast, eating in front of TV and eating fast and fried food. Further, the findings of this study highlight the importance of prioritizing intervention efforts for boys, children in socio-economically disadvantaged settings, and those with excess body weight as they are more likely to have unhealthy eating habits.

## Conclusions

This study identified three patterns of eating behaviours using LCA and observed a significant association of poor eating patterns with lower HRQoL in a sample of Canadian grade five students. Less and unhealthy eating patterns are associated with higher prevalence of obesity, lower diet quality and PA, lower levels of household income and parental education than the healthier eating pattern. The results confirm the previous findings showing that eating behaviours tend to cluster together with varying patterns, and the eating patterns are associate with a variety of health-related outcomes. The findings highlight the need to invest in effective interventions to improve healthy eating patterns in order to enhance HRQoL among children in this age group. School-based programs targeting healthy eating among children may be more effective if greater emphasis is given to subgroups of children with lower levels of healthful eating patterns rather than individual nutrient intake or single eating behaviour. Health promotion efforts focused on improving overall eating patterns may be easier than modifying individual nutrients or targeting isolated eating behaviours.

## Supplementary Information

Supplementary Tables.

## Data Availability

Study data for the analyses were secondary data. The source datasets presented in this paper are not readily available due to privacy and ethical restrictions. Requests to access the datasets should be directed to PJV, paul.veugelers@ualberta.ca.
