# Supplementary material for: The importance of eating patterns for health-related quality of life among children aged 10–11 years in Alberta of Canada
Source: Sci Rep. 2022 Dec 3;12:20885. doi: 10.1038/s41598-022-23707-7 (PMC9719497; doi:10.1038/s41598-022-23707-7)
Supplement: Supplementary file 1 — Supplementary Tables. [file 41598_2022_23707_MOESM1_ESM.docx]

**Supplementary Information**

S1 Table. Variables used for the eating patterns and their coding in the latent class analysis

| **Variable** | **Response level** | **Analytic coding** |
| --- | --- | --- |
| Skipping breakfast | No, Yes | 0 = No, 1 = Yes |
| Bringing prepared lunch from home | No, Yes | 0 = Yes, 1 = No |
| Buying lunch at schools | No, Yes | 0 = No, 1 = Yes |
| Fruit and vegetables intake | Number of daily servings | 0= ≥ 6 daily servings  1= < 6 daily servings |
| Buying snacks at schools | < 1 time per week  1-2 times per week  ≥ 3 times per week | 0 = < 1 time per week  1 = 1-2 times per week  2 = ≥ 3 times per week |
| Eating supper with family | ≤ 2 times per week  3-4 times per week  ≥ 5 times per week | 0 = ≥ 5 times per week  1 = 3-4 times per week  2 = ≤ 2 times per week |
| Eating supper in front of TV | < 1 time per month  1-2 times per week  ≥ 3 times per week | 0 = < 1 time per month  1 = 1-2 times per week  2 = ≥ 3 times per week |
| Eating supper alone | < 1 time per month  1-2 times per week  ≥ 3 times per week | 0 = < 1 time per month  1 = 1-2 times per week  2 = ≥ 3 times per week |
| Eating supper ready-made | < 1 time per month  1-2 times per week  ≥ 3 times per week | 0 = < 1 time per month  1 = 1-2 times per week  2 = ≥ 3 times per week |
| Eating at a fast food restaurant | < 1 time per month  1-2 times per week  ≥ 3 times per week | 0 = < 1 time per month  1 = 1-2 times per week  2 = ≥ 3 times per week |
| Eating fried food at home | < 1 time per week  1-3 times per week  4-6 times or daily per week | 0 = < 1 time per week  1 = 1-3 times per week  2 = 4-6 times or daily per week |
| Eating fried food outside home | < 1 time per week  1-3 times per week  4-6 times or daily per week | 0 = < 1 time per week  1 = 1-3 times per week  2 = 4-6 times or daily per week |

S2 Table. Fit statistics of the latent class models on the eating behaviour items, grade five children participating in the 2008, 2010 and 2012 Real Kids Alberta surveys Canada (n = 9150)

| **Classes** | **# Par** | **BIC** | **aBIC** | **LMRALRT p-value** | **Entropy** | **Class proportions** | | | | |
| --- | --- | --- | --- | --- | --- | --- | --- | --- | --- | --- |
|  |  |  |  |  |  | **Class 1** | **Class 2** | **Class 3** | **Class 4** | **Class 5** |
| 1-class | 20 | 140876.1 | 140812.6 | NA | NA | 1.00 |  |  |  |  |
| 2-class | 41 | 134759.9 | 134629.6 | < 0.0001 | 0.677 | 0.58 | 0.42 |  |  |  |
| **3-class** | **62** | **133461.7** | **133264.6** | **0.0235** | **0.664** | **0.52** | **0.31** | **0.17** |  |  |
| 4-class | 83 | 132728.0 | 132464.3 | 0.1087 | 0.724 | 0.50 | 0.31 | 0.14 | 0.05 |  |
| 5-class | 104 | 132144.2 | 131813.7 | 0.5121 | 0.725 | 0.43 | 0.31 | 0.13 | 0.09 | 0.04 |

# Par: number of free estimated parameters; BIC: Bayesian information criterion; aBIC: sample size adjusted BIC; LMRALRT: Lo-Mendell-Rubin adjusted likelihood ratio test. The class proportions were based on the classification of individuals based on their most likely latent class membership. The latent class analyses were weighted to accommodate the design effect such that the estimates represent the population of grade five children in the province of Alberta.
